# Impact of Day 7 Blastocyst Transfer on Obstetric and Perinatal Outcome of Singletons Born After Vitrified-Warmed Embryo Transfer

**DOI:** 10.3389/fphys.2020.00074

**Published:** 2020-02-12

**Authors:** Jiaan Huang, Xiaoyan Yang, Jiayi Wu, Yanping Kuang, Yun Wang

**Affiliations:** Department of Assisted Reproduction, Shanghai Ninth People’s Hospital Affiliated to Shanghai Jiao Tong University School of Medicine, Shanghai, China

**Keywords:** vitrified-warmed embryo transfer, day 7 blastocyst, obstetric outcome, perinatal outcome, singletons

## Abstract

**Background:**

Extended embryo culture has been reported to affect perinatal outcome regarding higher risks of large for gestational age (LGA) and preterm birth (PTB) yet decreased risk of small for gestational age (SGA). However, existing data about the obstetric outcome and the safety for offspring resulting from the transfer of day 7 blastocysts is rare.

**Objectives:**

To compare obstetric and perinatal outcome using embryos vitrified on day 7 with those vitrified on day 3, day 5, and day 6.

**Methods:**

Data were collected from 4489 infertile women who gave birth to live-born singletons after vitrified-warmed embryo transfer cycles from January 1, 2006 to December 31, 2017. Singletons were compared depending on the age of embryos. Main perinatal outcome parameters included PTB (gestational age < 37 weeks), very PTB (VPTB, gestational age < 32 weeks), LGA (birthweights > 90th percentiles), and SGA (birthweights < 10th percentiles). Obstetric outcomes included gestational diabetes (GDM), pregnancy-induced hypertension (PIH), preterm premature rupture of membranes (PPROM), pre-eclampsia, placenta previa, placental abruption, and postpartum hemorrhage. Propensity score matching (PSM) was used to adjust the confounding factors across groups and then analyze the association between *in vitro* culture period and the outcome measures.

**Results:**

After PSM, the transfer of day 7 blastocysts was associated with higher birth weight *Z*-scores and increased incidence of very large for gestational age (VLGA) compared with the transfer of day 3 cleavage-stage embryos while the incidence of PTB, low birth weight (LBW), SGA did not reach statistical significance. Moreover, comparable perinatal outcome was found in the comparison of day 7 vs. day 5 and day 7 vs. day 6. Day 7 blastocysts did not result in adverse obstetric outcome compared with day 3, day 5, and day 6 embryos, respectively.

**Conclusion:**

In vitrified-warmed transfer cycles, day 7 blastocysts were associated with adverse perinatal outcome regarding higher risk of VLGA compared with day 3 cleavage-stage embryo, while blastocysts with diverse growth rates embrace similar developmental viability regardless of blastocysts vitrified on day 5, day 6, or day 7.

## Introduction

Nowadays, *in vitro* fertilization (IVF) practice has presented a move toward blastocyst culture to achieve more favorable pregnancy outcomes compared to cleavage-stage embryo transfer (Glujovsky et al., 2016; Holden et al., 2018), especially since single embryo transfer policy has been advocating in many countries (Devine et al., 2015). Blastocyst culture is important for the selection of the most viable embryo for transfer, particularly with regards to reducing the incidence of multiple pregnancies, which increases the adverse obstetric and perinatal outcomes. Recently, an incremental increase of IVF cycles has been performed as vitrified-warmed embryo transfer with improved cryopreservation techniques (Pinborg et al., 2013), which played a key role in the refinement of blastocyst embryo transfer.

Over the past decades, embryos that do not reach blastulation on day 6 have previously been discarded in the practice of laboratory standard (Shoukir et al., 1998), however, the conventional practice of ceasing embryo culture on day 6 has been challenged by recent studies, which have demonstrated that embryos with delayed blastulation on day 7 can still be clinical viable (Morbeck, 2017; Hammond et al., 2018), reach top morphological grade (Whitney et al., 2019), achieve euploid status (Minasi et al., 2016; Whitney et al., 2019) and result in healthy newborns (Richter et al., 2016; Du et al., 2018). In general, embryos that did not develop into blastocysts on day 5 were considered with higher risk of aneuploidy and impaired viability, which would result in compromised pregnancy outcomes including implantation failure and reduction in live births, especially for day 7 blastocyst (Shapiro et al., 2008; Kovalevsky et al., 2013; Zhao et al., 2015; Richter et al., 2016; Wirleitner et al., 2016; Du et al., 2018; Hernandez-Nieto et al., 2019). Notably, there is growing evidence that euploid or cryopreserved day 7 blastocysts could result in lower but clinically valuable clinical pregnancy rate around 30–33% with sizable number of cases (Shapiro et al., 2008; Kovalevsky et al., 2013; Du et al., 2018; Hernandez-Nieto et al., 2019). However, there is a paucity of information comparing obstetric and perinatal outcome of day 7 blastocyst versus other stages of embryos, and the study end points have been mainly focused on pregnancy outcomes.

As we all know, the safe delivery of a healthy baby should be the prime goal of IVF. To date, conflicting data on the perinatal outcome of children have been reported after blastocyst transfer in comparison with cleavage-stage embryo transfer (Fernando et al., 2012; Makinen et al., 2013; Dar et al., 2014; Ishihara et al., 2014; Oron et al., 2014; Zhu et al., 2014; Zhang et al., 2019). There seems to be an improved perinatal outcome regarding lower odds of small for gestational age (SGA) yet increased risk of large for gestational age (LGA) and preterm birth (PTB) after blastocyst transfer (Dar et al., 2014; Ishihara et al., 2014). Moreover, the higher birthweight and *Z*-scores of singletons and higher proportion of LGA after extended embryo transfers have been found without an increase in PTB in some recent studies (Makinen et al., 2013; Zhu et al., 2014; Zhang et al., 2019), though others would not support it and indicated that *in vitro* culture period did not associate with increased adverse obstetric or perinatal outcome (Fernando et al., 2012; Oron et al., 2014). Nevertheless, these studies have been limited to inconsistent culture duration (day 2–4 for cleavage-stage and day 5–6 for blastocyst), and the different protocol of embryo transfer including fresh cycles and frozen embryo transfer (FET) cycles with slow-freezing or vitrification, which should also be considered responsible for the diverse outcomes.

As a result, there remains a matter of debate about the birth outcomes of day 7 blastocyst transfer, and it is a necessary exploration to assess the safety issue of those delayed embryo. The aim of our study was, therefore, to further analyze the obstetric and perinatal outcome of live-born singletons derived from day 7 blastocyst compared to those from day 3, day 5, and day 6 embryo transfers in vitrified-warmed cycles.

## Materials and Methods

### Study Population

We retrospectively evaluated 4489 infertile women who delivered live-born singletons derived from vitrified-warmed embryo transfer, after IVF or intracytoplasmic sperm injection (ICSI), between January 1, 2006 and December 31, 2017 at the Department of Assisted Reproduction of Shanghai Ninth People’s Hospital, Shanghai Jiao Tong University School of Medicine. The inclusion criteria were: (i) vitrified-warmed cycles with pure day 3, day 5, day 6, and day 7 embryo transfer, (ii) patients ≤ 40 years of age with a BMI ≤ 30 kg/m^2^, and (iii) the first singleton born alive after the 20th week of gestation with ART. Furthermore, patients with vanishing twin or without record delivery information were excluded, as was any use of PGD and assisted hatching. Women were included only once in the study.

### Ethics Statement

This study was approved by the Institutional Review Board of the hospital.

### Analysis and Propensity Score Matching

**FIGURE 1:**
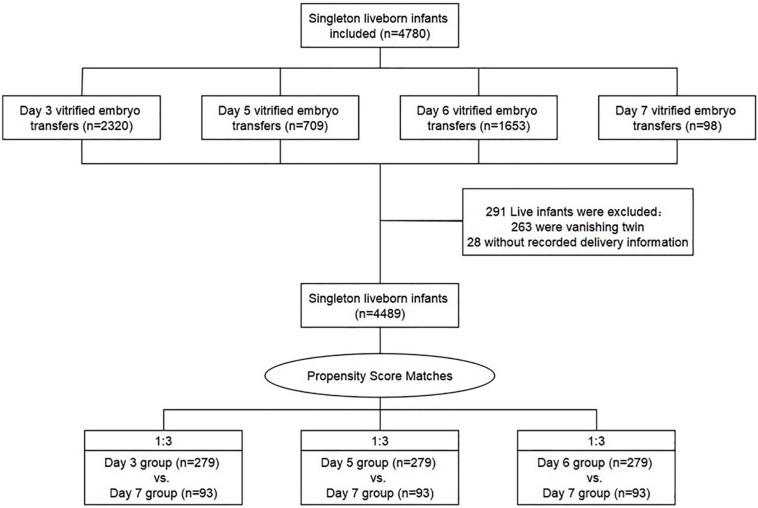
Flow chart of the study.

Following our inclusion criteria, 4489 cycles after vitrified-warmed embryo transfer have resulted in one live birth respectively: 2165 cycles with day 3 embryo transfer, 668 cycles with day 5 embryo transfer, 1563 cycles with day 6 embryo transfer, and 93 cycles with day 7 embryo transfer (Figure 1). Then, the method of propensity score matching (PSM) generated three matched cohorts (day 3 vs. day 7, day 5 vs. day 7, and day 6 vs. day 7), and the outcome parameters were compared between those matched groups (Austin, 2011). Propensity scores were output by logistic regression on the basis of patient and cycle characteristics including maternal age, BMI, duration of infertility, gravidity, parity, FET rank (first rank or higher rank), infertility causes (female, male, mixed, and other infertility type), endometrial preparation methods (natural cycles, stimulated cycles, and hormone therapy cycles), fertilization method (with ICSI or without ICSI), endometrial thickness on ET day, number of embryos transferred (one or two), and year of treatment (2006–2011, 2012–2013, and 2014–2017). We used a 1:3 matching algorithm of nearest neighbor random without replacement to obtained similar baseline covariates between the three groups.

### Laboratory Protocols

Details on protocols of controlled ovarian hyperstimulation, method of endometrial preparation and procedures of IVF/ICSI have been described previously (Chen et al., 2015; Du et al., 2018; Zhang et al., 2019). In brief, regarding semen analysis result and previous infertility characteristic, IVF or ICSI was performed 3–6 h after oocytes retrieval. For IVF, collected oocytes were incubated in human tubal fluid (HTF; Irvine Scientific, United States), supplemented with 10% serum substitute supplement (SSS; Irvine Scientific, United States), and 300,000 progressively motile spermatozoa and left overnight. For ICSI, denudated oocytes were injected with a single mechanically immobilized sperm and directly thereafter cultured in fertilization medium (HTF + 10% SSS). Notably, from 2013 onward, all embryos were maintained in Continuous Single Culture of HTF (Irvine Scientific, United States) throughout the entire duration of *in vitro* culture, however, before 2013, embryos were cultured in Early Cleavage Medium (Irvine Scientific, United States) before Day 3, and in MultiBlast Medium (Irvine Scientific, United States) sequentially. All embryos were incubated together under oil at 37°C and a 5% O_2_ and 6% CO_2_ humidified incubators with 30 μL of culture media drop. Eventually, Successful fertilization was indicated by the distinct appearance of two pronuclei 16–18 h after IVF/ICSI.

The process of blastocyst formation was decided by the overall quantity and quality of cleavage-stage embryos. After the observation by day 3, the morphology of embryos would be assessed and if the embryos were classified as poor quality (with <5 cells on day 3 and/or >20% fragmentation) (Reinblatt et al., 2011), the embryos would proceed blastulation. In the other case, when more than six cleavage-stage embryos are assessed as good quality, the supplementary cleavage-stage embryos will be assigned to blastulation utterly in spite of the embryo quality, and then they would be subsequently cultured and vitrified until they achieve blastocyst stage up to day 7. Additionally, the decision of embryos underwent day 7 blastulation and transfer was made by the request of patients and the evaluation of clinicians. The assessment of blastocyst quality was based on the scoring system of Gardner and Schoolcraft (1999). As a result, in all of the stimulated cycles included, there are around 8.5% of the cycles were intended for blastocysts transfer only. Of all blastocysts included in our study, the percent of embryos frozen in day 5, 6, and 7 were 26.9, 67.4, and 5.7%, respectively.

The vitrification process was performed using Cryotop carrier system combined with dimethylsulfoxide–ethylene glycol–sucrose as the cryoprotectants. During the vitrification procedure, one or two embryo(s) were placed on each single device. When the endometrial preparation was ready, the embryos were thawed via dilution solution in a sequential manner (1 mol/L to 0.5 mol/L to 0 mol/L sucrose) and then transferred back into uterus. For all embryos, including day 3, day 5, day 6, and day 7, the survival rate were over 99% in our study, and the time from warming to transfer was 3–4 h in groups.

### Outcome Measures

Perinatal outcomes evaluated were the followings: gestational age (calculated from the day of embryo transfer according to embryo stage and adjusted by ultrasonographic assessment, birthweight, *Z*-scores (birthweight after adjusted for gestational age and newborn gender), low birth weight (LBW, birthweights < 2500 g), very low birth weight (VLBW, birthweights < 1500 g), and high birth weight (HBW, birthweights > 4500 g), PTB (gestational age < 37 weeks) and very preterm birth (VPTB, gestational age < 32 weeks), SGA (birthweights < 10th percentiles) and very small for gestational age (VSGA, birthweights < 3rd percentiles), LGA (birthweights > 90th percentiles), and very large for gestational age (VLGA, birthweights > 97th percentiles). Obstetrics complications were classified in light of the International Classification of Diseases (ICD) Q codes (Q00–Q99) as conditions registered in the International Statistical Classification of Diseases and Related Health Problems, which included gestational diabetes (GDM; ICD 10 code O.24.4), pregnancy-induced hypertension (PIH; ICD 10 code O.13–14), preterm premature rupture of membranes (PPROM; ICD 10 code O.42.2–49.9), pre-eclampsia (ICD 10 code O.13–15), placenta previa (ICD 10 code O.44.0–44.1), placental abruption (ICD 10 code O.45), and postpartum hemorrhage (ICD 10 code O.72) in our study. Birthweight percentiles and calculation of *Z*-scores were based on Chinese reference singleton newborns stratified by gestational age and neonatal sex (Dai et al., 2014).

### Statistical Analyses

Statistical analysis was performed by SPSS version 24.0 software (SPSS Inc., Tsinghua, China). Continuous variables for patient baseline characteristics were assessed for normality graphically using the Shapiro–Wilk test combined with the frequency distribution curve and expressed as mean (±SD) or median (interquartile range) according to if variables were normally distributed or not. Categorical variables were presented with their frequency and percentage within the study group. After PSM, the baseline characteristic and outcome measures between-group were assessed using the *t*-test or Mann–Whitney–Wilcoxon tests for continuous variables, and comparisons of rates were performed by the Chi-square test or Fisher’s exact test as appropriate, and a Pearson correlation coefficient was provided for correlations. A *P*-value < 0.05 was considered to determine statistical significance.

## Results

**FIGURE 2:**
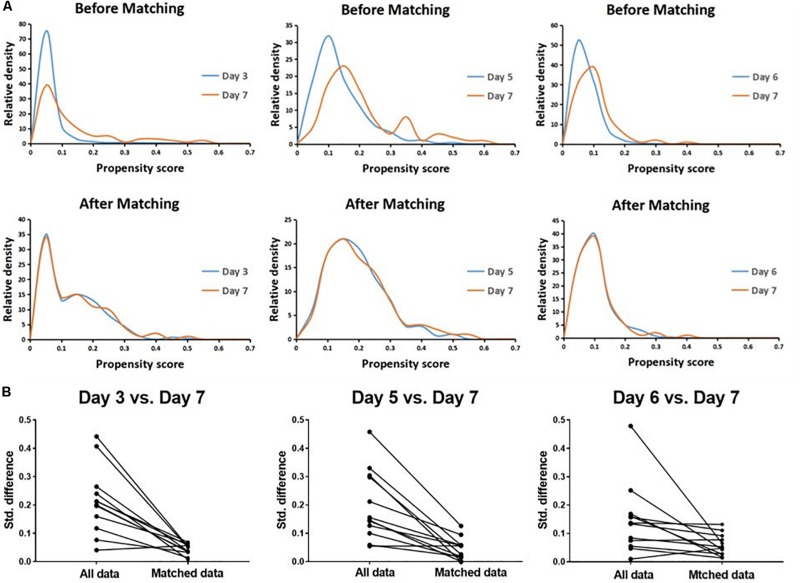
Propensity score matching (PSM) for day 3 vs. day 7, day 5 vs. day 7, and day 6 vs. day 7 cohorts, respectively. The relative density of propensity scores **(A)** and standard differences **(B)** before and after PSM showed balance between the compared cohorts.

A total of 4489 vitrified-warmed cycles from women who meet the inclusion criteria were included after the transfer of day 3, day 5, day 6, and day 7 embryo transfer, and they were further subdivided into three matched group (day 3 vs. day 7, day 5 vs. day 7, and day 6 vs. day 7) based upon PSM method to reach unbiased baseline characteristics (Figure 1). Every group comprised 279 cycles of transfers of day 3, day 5, or day 6 embryos, respectively, and 93 cycles of transfers of day 7 embryos according to the 1:3 matched ratio applied in all groups. The relative density of propensity scores and standard differences before and after PSM are illustrated in Figure 2, which showed the balance between the compared cohorts.

The pregnancy outcomes of day 3, day 5, day 6, and day 7 vitrified-warmed embryo transfer cycles are summarized in Table 1. Day 7 blastocyst shows significantly lower clinical pregnancy and live birth rate than day 3, day 5, and day 6 embryos. A significantly higher risk of miscarriage rate in the first trimester yet significantly lower ectopic rate was found in day 7 blastocysts compared with day 3 embryos. The ectopic rate and the miscarriage rate both in the first trimester and the second trimester did not reach statistical difference between day 5, day 6, and day 7 blastocysts. Besides, the stillbirth rate was similar between all groups.

**TABLE 1:** Pregnancy outcomes of day 3, day 5, day 6, and day 7 vitrified-warmed embryo transfer groups.

	Day 3 (*n* = 8591)	Day 5 (*n* = 1961)	Day 6 (*n* = 4910)	Day 7 (*n* = 413)	*P*-value^a^	*P*-value^b^	*P*-value^c^
Clinical pregnancies^d^	4313/8591 (50.2)	1194/1961 (60.9)	2685/4910 (54.7)	136/413 (32.9)	<*0.001*	<*0.001*	<*0.001*
Miscarriages	569/4313 (13.2)	174/1194 (14.6)	467/2685 (17.4)	26/136 (19.1)	0.046	0.160	0.605
1st trimester	496/4313 (11.5)	152/1194 (12.7)	414/2685 (15.4)	24/136 (17.6)	0.028	0.109	0.484
2st trimester	73/4313 (1.7)	22/1194 (1.8)	53/2685 (2.0)	2/136 (1.5)	0.843	0.758	0.679
Ectopic	125/4313 (2.9)	24/1194 (2.0)	21/2685 (0.8)	0/136 (0)	0.033	0.163	0.622
Live births^*d*^	3608/8591 (42.0)	994/1961 (50.7)	2193/4910 (44.7)	110/413 (26.6)	<*0.001*	<*0.001*	<*0.001*
Stillbirths	11/3608 (0.3)	2/994 (0.2)	4/2193 (0.2)	0/413 (0)	0.617	0.362	0.385

*Clinical pregnancy was diagnosed by the ultrasonographic visualization of one or more gestational sac after embryo transfer; Miscarriage rate was defined as a loss of clinical pregnancy before the 20th gestational week; Data are were presented as numbers and proportion (%). ^*a*^*P*-value for day 3 vs. day 7. ^*b*^*P*-value for day 5 vs. day 7. ^*c*^*P*-value for day 6 vs. day 7. ^*d*^Inclusion of single and twin pregnancies.*

Characteristics of the individual groups’ membership in matched vitrified-warmed embryo transfer groups are presented in Table 2, and all characteristics are distributed similarly between the matched groups.

**TABLE 2:** Patient and cycle characteristics of the matched day 3, day 5, day 6, and day 7 vitrified-warmed embryo transfer groups.

Characteristics	Day 3	Day 5	Day 6	Day 7	*P*-value^a^	*P*-value^b^	*P*-value^c^
Age (years)	32 (29, 34)	31 (28, 34)	32 (29, 35)	31 (29, 34)	0.876	0.601	0.931
BMI (kg/m^2^)	21.0 (19.5, 22.9)	20.8 (19.5, 22.6)	20.8 (19.5, 22.5)	20.7 (19.6, 22.6)	0.632	0.986	0.875
Duration of infertility (years)	3 (2, 5)	3 (2, 5)	3 (2, 5)	3 (2, 5)	0.570	0.741	0.785
Gravidity	0 (0, 1)	0 (0, 1)	0 (0, 1)	1 (0, 1)	0.643	0.837	0.723
Parity	0 (0, 0)	0 (0, 0)	0 (0, 0)	0 (0, 0)	0.901	0.918	0.845
FET rank							
First	196 (70.3)	195 (69.9)	183 (65.6)	65 (69.9)	0.948	1	0.446
Higher rank	83 (29.7)	84 (30.1)	96 (34.4)	28 (30.1)			
Main infertility cause (%)					0.647	0.882	0.830
Female	145 (52.0)	157 (56.3)	157 (56.3)	50 (53.8)			
Male	47 (16.8)	29 (10.4)	38 (13.6)	11 (11.8)			
Mixed	67 (24.0)	80 (28.7)	65 (23.3)	26 (28.0)			
Other	20 (7.2)	13 (4.7)	19 (6.8)	6 (6.5)			
Endometrial preparation (%)					0.526	0.887	0.440
Natural cycles	64 (22.9)	71 (25.4)	87 (31.2)	26 (28.0)			
Hormone therapy cycles	121 (43.4)	107 (38.4)	85 (30.5)	35 (37.6)			
Stimulated cycles	94 (33.7)	101 (36.2)	107 (38.4)	32 (34.4)			
Cycles with ICSI (%)	127 (45.5)	129 (46.2)	123 (44.1)	43 (46.2)	0.904	1	0.718
Endometrial thickness on ET day (mm)	10.3 (9.1, 11.9)	10.3 (9.2, 11.8)	10.5 (9.1, 12.1)	10.2 (9.1, 12.3)	0.738	0.797	0.872
No. of embryo transferred					0.673	0.630	0.904
1	157 (56.3)	158 (56.6)	148 (53.0)	50 (53.8)			
2	122 (43.7)	121 (43.4)	131 (47.0)	43 (46.2)			
Year of treatment (%)					0.770	0.438	0.863
2006–2011	74 (26.5)	66 (23.7)	77 (27.6)	28 (30.1)			
2012–2013	75 (26.9)	87 (31.2)	79 (28.3)	25 (26.9)			
2014–2017	130 (46.6)	126 (45.2)	123 (44.1)	40 (43.0)			

*FET, frozen embryo transfer. ET, embryo transfer. Data are presented as mean (±SD) or median (first quartile, third quartile) according to if variables were normally distributed or not. Continuous variables were compared using *t*-test or Mann–Whitney–Wilcoxon tests, and categorical variables were presented as numbers (%) and evaluated with Chi-squared tests. ^*a*^*P*-value for day 3 vs. day 7. ^*b*^*P*-value for day 5 vs. day 7. ^*c*^*P*-value for day 6 vs. day 7.*

Descriptive statistics for the obstetric and perinatal outcomes according to embryo culture length are shown in Table 3. The median and interquartile range of birthweight were 3350 (3020, 3610) g, 3400 (3068, 3700) g, 3340 (3050, 3690) g, and 3400 (3095, 3665) g in the groups of day 3, day 5, day 6, and day 7, respectively, and no significant differences were observed (day 7 vs. day 3: *P* = 0.249; day 7 vs. day 5: *P* = 0.945; and day 7 vs. day 6: *P* = 0.441, respectively). However, a higher *Z*-score was detected in the day 7 group than in the day 3 group (0.55 (−0.22, 1.30) vs. 0.27 (−0.41, 0.94), *P* = 0.027). Also, an increased risk of VLGA were found in the day 7 group than in the day 3 group (12.9% vs. 6.1%, *P* = 0.034), while a higher rate of PTB (7.6% vs. 3.9%, *P* = 0.170) and a lower rate of SGA (2.2% vs. 5.7%, *P* = 0.262) was found in the group of day 7, though the difference was not statistically significant. The rate of LBW (2.2% vs. 2.5%, *P* = 0.846) was also comparable between the matched groups of day 7 and day 3. For the matched groups of day 5 vs. day 7 and day 6 vs. day 7, slightly higher rates of LGA and VLGA, and lower rate of SGA were found in the day 7 group in comparison to the groups of day 5 and day 6, not reaching statistically significant. The other perinatal measures, including the proportion of VPTB, VLBW, HBW, LGA, and VSGA were similarly distributed in all matched groups.

**TABLE 3:** Obstetric and perinatal outcomes of the matched day 3, day 5, day 6, and day 7 vitrified-warmed embryo transfer groups.

Outcome	Day 3	Day 5	Day 6	Day 7	*P*-value^a^	*P*-value^b^	*P*-value^c^
Gestational age (weeks)							
<32 weeks	2 (0.7)	0 (0.0)	3 (1.1)	1 (1.1)	0.579	0.250	1
32–36 weeks	9 (3.2)	19 (6.8)	15 (5.4)	6 (6.5)	0.220	0.905	0.697
≥37 weeks	268 (96.1)	260 (93.2)	261 (93.5)	86 (92.5)	0.170	0.814	0.720
Mode of delivery (%)					**0.027**	0.198	0.883
Vaginal	94 (33.7)	79 (28.3)	58 (20.8)	20 (21.5)			
Cesarean section	185 (66.3)	200 (71.7)	221 (79.2)	73 (78.5)			
Sex (%)					0.905	0.588	0.469
Male	145 (52.0)	156 (55.9)	159 (57.0)	49 (52.7)			
Female	134 (48.0)	123 (44.1)	120 (43.0)	44 (47.3)			
Birthweight (g)	3350 (3020, 3610)	3400 (3068, 3700)	3340 (3050, 3690)	3400 (3095, 3665)	0.249	0.945	0.441
*Z*-score	0.27 (−0.41, 0.94)	0.42 (−0.22, 1.21)	0.32 (−0.34, 1.11)	0.55 (−0.22, 1.30)	**0.027**	0.707	0.215
<1500 g	2 (0.7)	1 (0.4)	3 (1.1)	0 (0.0)	0.562	0.750	0.576
<2500 g	7 (2.5)	9 (3.2)	11 (3.9)	2 (2.2)	0.846	0.738	0.531
2500–4500 g	271 (97.1)	266 (95.3)	265 (95.0)	88 (94.6)	0.325	0.783	0.892
>4500 g	1 (0.4)	4 (1.4)	3 (1.1)	3 (3.2)	0.050	0.373	0.168
SGA (<10th percentile)	16 (5.7)	13 (4.7)	10 (3.6)	2 (2.2)	0.262	0.374	0.738
VSGA (<3th percentile)	5 (1.8)	4 (1.4)	3 (1.1)	1 (1.1)	0.635	0.795	0.739
LGA (>90th percentile)	48 (17.2)	64 (22.9)	53 (19.0)	22 (23.7)	0.168	0.887	0.332
VLGA (>97th percentile)	17 (6.1)	30 (10.8)	27 (9.7)	12 (12.9)	**0.034**	0.570	0.379
Pregnancy complication (%)							
GDM	35 (12.5)	28 (10.0)	39 (14.0)	10 (10.8)	0.646	0.843	0.426
PIH	14 (5.0)	11 (3.9)	12 (4.3)	3 (3.2)	0.579	0.753	0.770
PPROM	8 (2.9)	5 (1.8)	4 (1.4)	4 (4.3)	0.504	0.236	0.111
Pre-eclampsia	7 (2.5)	6 (2.2)	3 (1.1)	1 (1.1)	0.685	0.685	0.586
Placenta previa	2 (0.7)	5 (1.8)	6 (2.2)	3 (3.2)	0.102	0.418	0.696
Placental abruption	2 (0.7)	2 (0.7)	1 (0.4)	1 (1.1)	0.738	0.738	0.438
Postpartum hemorrhage	6 (2.2)	8 (2.9)	10 (3.6)	2 (2.2)	1	0.711	0.675

*PIH, pregnancy-induced hypertension; PPROM, preterm premature rupture of membranes. ^*a*^*P*-value for day 3 vs. day 7. ^*b*^*P*-value for day 5 vs. day 7. ^*c*^*P*-value for day 6 vs. day 7. Bold values indicate statistically significant results.*

Regarding obstetric outcome, slightly higher incidence of placenta previa was found in day 7 group (3.2%) compared with matched day 3 (0.7%), day 5 (1.8%), and day 6 group (2.2%), though no significant difference was detected (Table 3). We also evaluated the associations between embryo culture duration and the incidence of GDM, PIH, PPROM, pre-eclampsia, placental abruption and postpartum hemorrhage, but no significant difference was found.

## Discussion

This is the first study exploring the impact of day 7 blastocysts on obstetric and perinatal outcomes of singletons born after vitrified-warmed embryo transfer cycles. Our results demonstrated that day 7 blastocysts were associated with higher *Z*-score and higher risks of VLGA compared with day 3 group, and we did not found any significant difference in the comparison between day 5, day 6, and day 7 group. The pregnancy outcomes of day 3, day 5, day 6, and day 7 vitrified-warmed embryo transfer cycles was in accordance with previous studies (Du et al., 2017, 2018; Zhu et al., 2019), that day 7 blastocyst transfer would result in lower but still acceptable live birth rate. In addition, day 7 blastocyst transfer was associated with a significantly lower risk of ectopic pregnancy compared with day 3 embryo transfer (Du et al., 2017).

In line with previous studies, which have been repeatedly described that the higher birthweight or *Z*-score and higher risk of LGA and VLGA existed after blastocyst transfer compared with cleavage-stage embryo transfer in FET cycles (Makinen et al., 2013; Ishihara et al., 2014; Zhu et al., 2014; Zhang et al., 2019), our findings showed the association between day 7 and day 3 embryo transfer and focused the main problem in VLGA. This result would raise concern about long-term safety after the transfer of day 7 blastocyst, including diabetes mellitus and cardiovascular disease (Belbasis et al., 2016). However, regarding the comparison between day 5, day 6, and day 7, the comparable outcome between groups indicated that all stages of blastocysts embraced similar developmental potentials on fetus and seem to be relatively reliable for the offspring. This is in accordance with the existing literature data which have referred to the neonatal outcome of day 7 blastocysts (Hiraoka et al., 2009; Du et al., 2018). One study has investigated the risks of LBW, early neonatal death and congenital malformation of live-born infants after day 7 blastocyst transfer compared with those born after day 5 and day 6 blastocyst, and the no significant difference was observed in the adverse neonatal outcome, thought the study did not adjust for potential confounders (Du et al., 2018). The other study did not found any difference in the mean gestational age, the incidence of PTB, and mean birthweight between day 5, day 6, and day 7 blastocysts transfer, suggesting that all blastocyst have homologous inherent viability, however, the sample size was exceeding low (*n* = 47 for day 5; *n* = 26 for day 6; and *n* = 8 for day 7) and confounded by vitrified embryos transferred with mix of different days and the presence of twins (Hiraoka et al., 2009).

Some recent studies have reported that FET was associated with better obstetric and perinatal outcomes regarding lower risks of LBW, PTB, SGA, placenta previa and placental abruption yet increased PIH, placenta accreta and postpartum hemorrhage compared with fresh embryo transfer (Ishihara et al., 2014; Martin et al., 2017; Sha et al., 2018), which may attributed to the physical effect of cryopreservation to filter out weaker embryos with inferior implantation and growth potential. In this case, the procedure of vitrification used in our study may have a positive impact on the embryo transfer leading to decreased adverse neonatal and maternal risks. On the other side, several observational studies have found an increase in the risk of placenta previa in pregnancies after blastocysts transfer compared with cleavage-stage embryo transfer (Ginstrom Ernstad et al., 2016), but the proportion of obstetric complications did not vary between compared groups in our study. However, our current findings should be interpreted with caution to take a form conclusion on obstetric outcome, due to the relatively small number of day 7 blastocysts transfer (*n* = 93) and low prevalence of maternal complications in the study population.

It has been suggested that several confounders, including maternal age, infertility cause, lifestyle factors, as well as laboratory conditions: the culture media, temperature, pH, CO_2_, and O2 concentration during the embryo culture, additional embryo manipulation, and also extended culture to day 7 which is investigated in this study, might induce epigenetic changes and therefore have an effect on placentation and finally on the obstetric and perinatal outcomes. Notably, the embryo culture media was changed after 2013 in our institution, by using the continuous embryo culture media rather than sequential media, we would be able to reduce any stress during the culture, and maintain culture conditions steady to promote the preimplantation development of embryos and elicit higher blastulation (Alhelou et al., 2018; Cimadomo et al., 2018). In addition, the Cryotop system used during the procedure of vitrification would result in slightly higher survival and developmental rates of embryos compared with closed system, though the difference did not reach significance (Kuwayama et al., 2005). Given that the open system had direct contact with nitrogen, the liquid nitrogen would be better to be sterilized to eliminate the risk of embryo contamination. Studies have suggested to sterilize liquid nitrogen using ultraviolet light or storage in vapor phase liquid nitrogen, which contains a lower density of contaminants (Bielanski et al., 2000; Bielanski, 2012).

Furthermore, considering the higher risk of VLGA after day 7 blastocyst compared with day 3 embryo transfer, a recent study focused on LGA infants have suggested that comparing to infants born with appropriate birthweight, the morbidity of LGA was related to the alteration of DNA methylation pattern in genes at the early embryonic stages, which would bring into cardiometabolic risk in children, as a results of anomalous placentation and overgrowth of the fetus (Lin et al., 2016). Additionally, the large offspring syndrome observed in animals has been attributed to the exposure to *in vitro* culture, with aberration in the expression of multiple growth-related imprinted genes (Young et al., 1998). Several studies speculated that prolonged exposure to the external environment might predispose human embryos to imprinting disorders and the alteration of genomic epigenetic modification that may persist throughout fetal development until birth, resulting in increased fetal size (Zhu et al., 2014; Kleijkers et al., 2016). However, the results of other existing studies could not confirm the explanation (Fernando et al., 2012; Oron et al., 2014; De Vos et al., 2018).

Day 7 blastocyst could reach maturation with prolonged time after insemination and result in suboptimal ratio of inner cell mass (ICM) and trophectoderm (TE) to some extent (Campbell et al., 2013; Martin et al., 2017), namely increased proportion of inferior morphological grades, which may result in an underlying pathology of worse prognosis concerned with poor embryo quality. Similarly, those growth-retarded blastocysts was also concerned with damaged embryonic euploid viability (Capalbo et al., 2014; Irani et al., 2019), though our study did not found any difference in the risks of PTB, LBW, SGA, and the other adverse perinatal outcome after day 7 blastocysts transfer compared with matched groups of day 3, day 5, or day 6, which indicating that the negative effect of aneuploidy and poor embryo quality may mainly realize in failed implantation and clinical loss, while less resulted in affected babies (Capalbo et al., 2014; Morbeck, 2017; Du et al., 2018; Irani et al., 2019). Above all, in the interest of IVF strategy, in spite of the stage of embryos transferred or the cryopreservation protocols, it is to be remembered that we ultimately aimed for achieving a healthy live baby thus would stand a promising chance to grow into a healthy adult later in life.

In addition, it is worth noting that, in the current study, the PSM method was used to minimize the effects of confounding for maximum equilibrium among day 3, day 5, day 6, and day 7 vitrified-warmed embryo transfers, thus to mimic some of the initial characteristics of prospective study (Austin, 2011). After PSM, we obtained similar baseline covariates between the matched groups of each set of two (Figure 1 and Table 1), which made our finding more convincing. Moreover, our study included 93 live-born singletons derived from day 7 vitrified-warmed embryo transfer, which is the largest number reported on this topic so far, providing a valuable chance to evaluate the impact of these relatively uncommon embryos.

Our study was mainly limited by the number of day 7 blastocysts transfer and the retrospective assessment, which including the absence of some information on potential confounders such as maternal smoking status, but the proportion of smoking women was quite few in China^1^.

## Conclusion

In summary, the ultimate analysis of *in vitro* culture period is the promise of a healthy pregnancy that progresses to a live birth. Our findings could help patients when they consult clinicians on the chance of delivering a healthy normal-weight baby after day 7 blastocysts transfer, and our results indicating that blastocysts with diverse growth rates would embrace similar developmental viability regardless of blastocysts vitrified on day 5, day 6, or day 7.

The exploration of IVF and cryopreservation program is always on its way of practice. The impact of day 7 blastocyst warrants further investigation by larger registry study to obtain a more accurate understanding of the association between embryo of delayed blastulation and obstetric and perinatal outcome.

## Data Availability Statement

All datasets generated for this study are included in the article/supplementary material.

## Ethics Statement

The studies involving human participants were reviewed and approved by the Shanghai Ninth People’s Hospital Institutional Review Board. The patients/participants provided their written informed consent to participate in this study.

## Author Contributions

YW supervised the entire study, including the procedures, conception, design, and completion. XY, JW, and YK were responsible for the collection of the data. JH contributed the data analysis and drafted the manuscript. YW participated in the interpretation of the study data and in revisions to the manuscript.

## Conflict of Interest

The authors declare that the research was conducted in the absence of any commercial or financial relationships that could be construed as a potential conflict of interest.

## Footnotes

1 http://news.xinhuanet.com/fortune/2013 –05/31/c_124794069.htm
